# Modified balloon-stent kissing technique avoid side-branch compromise for simple true bifurcation lesions

**DOI:** 10.1186/s12872-019-1052-0

**Published:** 2019-04-08

**Authors:** Wen-Bo Qu, Wei Zhang, Jun-Yan Liu, Fan Zhang, Shuai-Nan Mu, Sheng-Ming Zhang, Hao Tang, Xi-Qian Liu, Xue-Qi Li, Bing-Chen Liu

**Affiliations:** Department of Cardiology, The Fourth Affiliated Hospital of Harbin Medical University, Harbin, Heilongjiang 150086 People’s Republic of China

**Keywords:** Modified balloon-stent kissing technique, Provisional stenting, Bifurcation lesions, Percutaneous coronary intervention, Side branch deterioration, Acute coronary syndrame

## Abstract

**Background:**

Coronary bifurcation remains one of the most challenging lesion subsets in interventinal cardiology. Provisional stenting (PS) is the dominate technique for bifurcation lesions, but the key problem is the deterioration of side branch. Balloon-stent kissing technique (BSKT) as a new systematic approach which is based on modified jailed balloon technique is applied to improve the procedure success. In our center, we proposed a modified balloon-stent kissing technique(M-BSKT), which routine usage of proximal optimizing technique (POT) after rewiring was added as an optimization step to BSKT. Thus, whether M-BSKT for addressing simple true coronary bifurcation lesions can provide more benefits in intra-operation effect and long term outcomes is still unknown.

**Methods:**

A cohort of 120 consecutive patients underwent Percutaneous Coronary Intervention (PCI) with simple true coronary bifurcation lesions satisfied the criteria were included in this retrospective, single-center registry. To assemble a cohort with similar baseline characteristics, a 1:1 propensity-matched score was used. The primary outcomes were the rate of device and procedural success, the situation of side branch (SB) after main vessel (MV) inflation and the complications during intra-operative. The secondary outcomes were the clinical prognosis at 12 months such as rehospitalization for unstable angina and MACEs.

**Results:**

Before propensity matching, there were no significant differences in primary and secondary outcomes between two groups. After propensity-matched was used, 68 patients with similar propensity scores were included. At immediate procedural, M-BSKT was associated with a lower risk of SB deterioration and the application of final kissing balloon inflation (FKBI)[*P* = 0.036]. For ACS patients, besides the significant differences of immediate SB deterioration [*P* = 0.014] and FKBI application [*P* = 0.033], the incidence of TIMI flow< 3 in the PS was statistically significant higher than M-BSKT [*P*= 0.042]. The prognosis at 12 months such as rehospitalization for unstable angina and MACEs were similar for two groups [*P* = 0.613].

**Conclusion:**

These observations prove that the M-BSKT enables side branch to be better protected in simple true bifurcation lesions, by a narrow margin. It may improve the angiographic outcomes about side branch deterioration and final kissing balloon performing compared with PS, especially in ACS patients. However, long-term clinical outcomes did not differ between patients treated for M-BSKT and PS at 12 months.

## Background

Coronary bifurcations are frequent and account for approximately 20% of all percutaneous coronary interventions [1]. Nonetheless,they represent one of the remaining challenges in interventional cardiology and the uniform strategy is still the subject of substantial debate in terms of a lower procedural success rate,a higher risk of procedural complications, and increasing rates of long-term adverse cardiac events [2].

During stent implantation, provisional stenting (PS) was often performed, where a conventional guide wire was inserted to the side branch (SB) before implanting stent to the main vessel (MV), if it wasn’t effective, then the SB could be treated. It has been widely accepted as the gold standard for its lower risks of major adverse cardiac events (MADEs), death, myocardial infarction (MI), and target vessel revascularization (TVR) in the majority of bifurcation lesions [3–11].

Whereas, simple strategy may shift the carina to the SB and induce a stenosis after stenting of MV. Major SB occlusion after MV stenting is one of the most serious complications closely associated with cardiac death and MI, and it may be the major reason why operators prefer more aggressive strategy in the bifurcation lesions [12–15]. Therefore, to prevent from carina displacement—the basic mechanism of side branch compromise during bifurcation percutaneous coronary intervention, several novel stenting systems for bifurcation lesions have been developed [16, 17].

The main purpose of this study was to perform a novel SB protection technique called Modified balloon-stent kissing technique (M-BSKT), which was based on balloon-stent kissing technique that was first proposed by Jin Z [18]. Routine usage of proximal optimizing technique (POT) after rewiring could make the malapposition in the stented MV segment completely corrected while maintaining perfect arterial circularity and achieving effective modification of physiological anatomy [19]. With this improvement, we modified balloon-stent kissing technique and discussed the advantage compared to other SB protection techniques such as PS.

## Methods

### Study population

From January 2015 to July 2017, a cohort of 120 consecutive patients who defined as true bifurcation lesions undergoing PCI were collected at The Fourth Affiliated Hospital of Harbin Medical University, Harbin, China.

All the patients enrolled in our study population were patients with simple true bifurcation lesions (excluding the complex bifurcation lesions [20]). The study was approved by the institutional review board, and informed consents for participation in the trial were obtained from all patients.

Data collection and monitoring were performed by an independent third-party contract research organization. The executive committee met regularly in person to monitor all aspects of the conduct of the trial.

#### Inclusion criteria

1. Patients were eligible for the studies if they were 18 to75 years old with true coronary bifurcation lesions undergoing PCI
2. The true bifurcation lesion consisted at least one major SB, bifurcation classifications were made according to Medina classification [21]. Medina 1,1,1 1,0,1 and 0,1,1 coronary bifurcation lesions with an SB diameter ≥ 2.0 mm based on visual estimation were included in the training and study groups

#### Exclusion criteria

1. The bifurcation lesion was categorized as complex bifurcation lesions according to the DEFINITION [20], defined as Medina 1,1,1and 0,1,1 coronary bifurcation lesions with each major criterion (left main vessel with ostial SB lesion length ≥ 10 mm and diameter stenosis (DS) ≥ 70%; non-left main vessel with ostial SB lesion length ≥ 10 mm and DS ≥ 90%) plus any 2 minor criteria (moderate to severe calcification; multiple lesions; bifurcation angle >45; main vessel RVD<2.5mm; thrombus-containing lesions; MV lesion length ≥25mm)
2. Subject with renal failure (serum creatinine > 2.0 mg/dl)
3. Subject exhibited severe left ventricular dysfunction (left ventricular ejection fraction < 35%)
4. Subject with a serious comorbidity or with life expectancy < 1 year
5. Subject exhibited contraindications to aspirin or clopidogrel

#### Procedure and outcomes

According to the ratio of 1:2, we randomly gathered patients with simple true bifurcation lesions to M-BSKT (*n* = 40) and PS (*n* = 80). The study patients in both groups received the same routine preparation with antiplatelet and antithrombotic medications based on the current guidelines. Coronary angioplasty was performed in conventional manner, and the interventional strategy and instrumentation used were at the discretion of the interventional cardiologists. Decisions on treatment strategy for bifurcation lesions were attempted according to the randomization process by individual operators.

##### Provisional stenting (jailed wire) technique

The recommended strategy is to wire in both MV and SB, while the lesion preparation is made at the operator’s discretion. The MV is stented with jailed wire protecting in SB. Proximal Optimization Technique (POT) is mandated to achieve good apposition of the proximal MV stent after the SB is rewired with or without rescue final kissing (Indications are shown below). The wire in SB will not be removed until the POT is completed.

##### Modified balloon-stent kissing technique

**Fig. 1:**
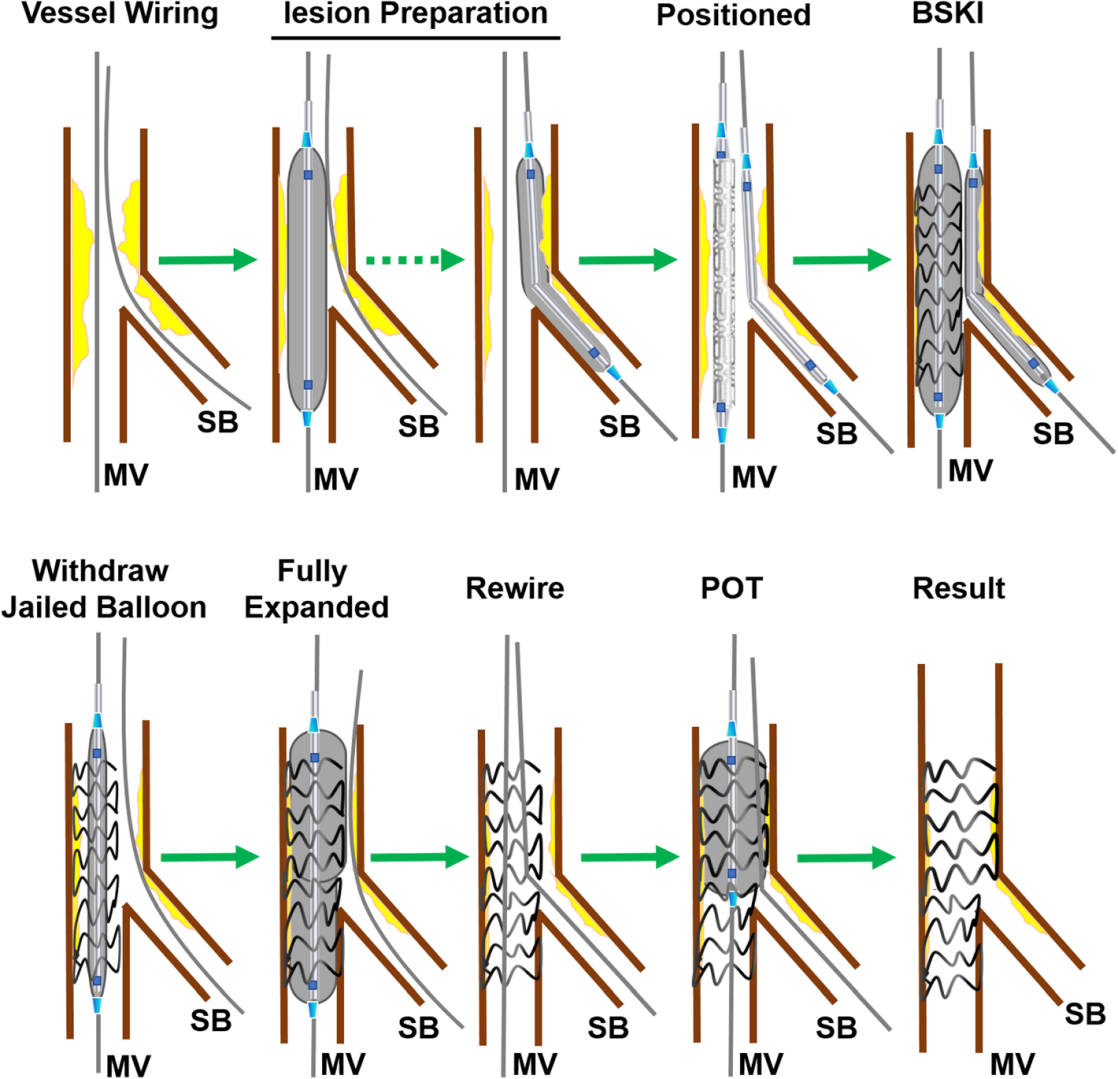
The details of modified balloon-stent kissing technique. BSKI: balloon-stent kissing inflation; MB: main branch; SB: side branch; POT: proximal optimization technique

**Fig. 2:**
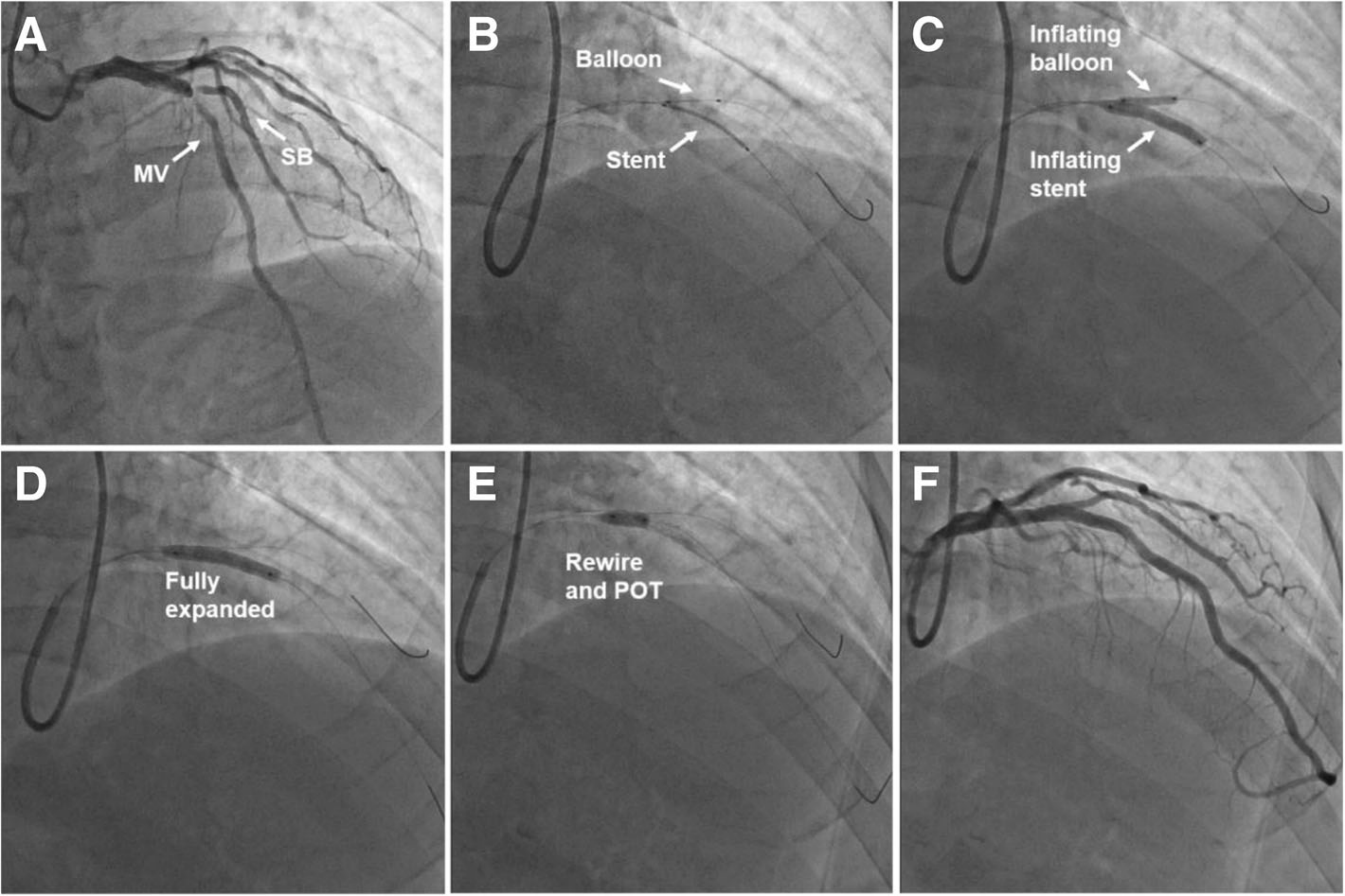
Coronary angiography illustrating the modified balloon-stent kissing technique. **a**: The angiogram demonstrates severe stenosis at simple true bifurcation segment (Medina 1,1,1). **b**: A balloon that is sized to the reference vessel diameter of SB (2.5 mm) is advanced into the SB. A stent (3.5 mm) is then advanced into correct position over the target lesion in the MV. **c**: The jailed SB balloon is inflated to 6 atm, meanwhile, the stent in MV is deployed to 8 atm. **d**: The stent balloon is fully expanded to 12 atm. **e**: The SB is rewired followed by POT (4.0 mm non-compliant balloon). **f**: No residual stenosis both in MV and SB in final angiogram

The initial technique has been detailed in previous study [18]. The M-BSKT used at our institution is detailed in Fig. 1 and shown angiographically in Fig. 2: To be brief, vessel wiring and lesion preparation are the same as the jailed wire technique. A balloon that is appropriately sized to approximate or smaller than the reference vessel diameter of SB (generally 1.5–2.5 mm) is advanced into the SB. A stent is then advanced into correct position over the target lesion in the MV. Adequate length of balloon is advanced into SB to project the ostium. Then the jailed SB balloon is first inflated to low pressure (6-8 atm), meanwhile, the stent in MV is deployed to nominal pressure (8-10 atm)(Fig. 2b-c). The SB balloon is removed and the wire remains in SB. The stent balloon is then fully expanded to moderate or high pressure as clinically indicated for the optimization of the stent full expansion at the distal MV, and to correct any stent deformation as a result of the SB balloon inflation (Fig. 2d). If the SB is not compromised, the SB will be rewired followed by POT (Fig. 2e) that achieve effective modification of physiological anatomy. Final result was good in showing case (Fig. 2f).

The SB is not further treated both in PS and M-BSKT groups unless there is threatened SB closure, severe ostial pinching of SB (> 90%), TIMI flow grade decrease in SB, or SB dissection greater than type A. If one of these criteria exists, the SB will be rewired and a final kissing balloon inflation (FKBI) will be undertaken with anatomically appropriate sizing for each vessel. A bailout stent in side branch is implanted only in case of dissection or significant flow impairment after FKBI. Finally, the re-POT strategy is mandated to achieve optimization apposition of the proximal MV stent. In addition, it needs to be emphasized that the MV stent should be sized according to the distal main vessel diameter to prevent irreversible carina shift, and the non-compliant balloon should be chosen for POT and sized according to the Murray’s law.

At postprocedure, all patients received aspirin (100 mg/d) indefinitely, and clopidogrel (75 mg/d) would be recommended for at least 12 months. The administration of glycoprotein IIb/IIIa inhibitors was performed at the discretion of the operator.

#### Follow-up

Clinical follow-up was performed by physicians via medical chart review or telephone interview at 12 months. Adverse events were monitored throughout the entire study period. Angiographic re-evaluation was planned after 1 year. The information of 120 patients was eventually collected.

#### Primary and secondary endpoint(s)

The primary endpoints of the study were the rates of device and procedural success (lesion success without the occurrence of in-hospital major adverse cardiac events (MACEs) such as death, myocardial infarction (MI), emergent coronary artery bypass graft (CABG), clinically driven target lesion revascularization (TLR)), the total numbers of FKBI used (because of the SB ostial pinching ≥90% and the TIMI flow< 3 after the MB stenting), the incidence of SB occlusion(after MV stent inflation) and the probability of balloons and wires damaged or even broken during their removal.

Secondary endpoints were a composite of the rehospitalization rate due to angina pectoris and MACEs at 12 months (defined as cardiac death, MI, and any TLR by PCI or CABG).

### Statistical analysis

Descriptive statistics were presented as percentages for categorical variables. Baseline characteristics were compared using a Chi-square or Fisher exact test for categorical variables and a Mann-Whitney U test for continuous variables because of they not compared to normal distribution. For non-normally distributed variables, median and IQR were used. In addition to conventional multivariate analysis, we constructed a propensity score within a ± 0.2 caliper range for adjustment and matching using R package matching. Matched categorical and continuous variables were presented same as before. A *P* value of < 0.05 was considered to represent a statistically significant difference. All the statistical models were performed using R Programming Language.

## Results

### Baseline characteristics

Between January 2015 and July 2017,a total of 120patients with true coronary bifurcation lesions satisfied all inclusion/exclusion criteria undergoing PCI were enrolled and ramdomly assigned to M-BSKT(*n* = 40) and PS(*n* = 80). All patients were followed up for 12 months. There were no significant differences between the two groups for patient’s baseline clinical characteristics (Table 1) and lesion characteristics (Table 2),respectively.

**Table 1:** Baseline Characteristics

	pre propensity-matched analysis			post propensity-matched analysis		
	M-BSKT	PS	*P* Value	M-BSKT	PS	*P* Value
	n = 40	n = 80		*n* = 34	n = 34	
Male	30 (75%)	62 (77.5%)	0.939	25 (73.5%)	26 (76.5%)	1
Hypertension	28 (70%)	42 (52.5%)	0.102	22 (64.7%)	21 (61.8%)	1
Diabetes Mellitus	14 (35%)	19 (23.8%)	0.202	10 (29.4%)	10 (29.4%)	1
Hyperlipidemia	14 (35%)	25 (31.3%)	0.836	13 (38.2%)	10 (29.4%)	0.608
Smoker	15 (37.5%)	39 (48.8%)	0.331	14 (41.2%)	20 (58.8%)	0.225
prior CABG	1 (2.5%)	0	0.333	0	0	NS
prior PCI	6 (15%)	14 (17.5%)	0.931	5 (14.7%)	3 (8.8%)	0.709
prior MI	4 (10%)	9 (22.5%)	1	2 (5.9%)	1 (2.9%)	1
Chronic kidney disease	1 (2.5%)	2 (2.5%)	1	1 (2.9%)	0	1
Diagnosis at PCI						
STEMI	3 (7.5%)	11 (13.8%)	0.464	2 (5.9%)	4 (11.7%)	0.577
NSTEMI	9 (22.5%)	22 (27.5%)		6 (17.6%)	8 (23.5%)	
Angina	28 (70%)	47 (58.8%)		26 (76.5%)	22 (64.7%)	
Age	62	59.5	0.255	62	59	0.368
IQR	12	13		13.5	10.5	
TNI	0.01	0.01	0.487	0.01	0.01	0.533
IQR	0.068	0.187		0.021	0.21	
CK-MB	14	15	0.397	14	15	0.1
IQR	4.25	6.25		4	5.75	

Data are presented as median (interquartile range) for continuous variables and n (%) for categorical variables. Chi-square or Fisher exact test for categorical variables and a Mann-Whitney U test for continuous variables. CABG, coronary artery bypass graft; PCI, percutaneous coronary intervention; MI, myocardial infarction; STEMI, ST-segment elevation myocardial infarction; NSTEMI, non-ST-segment elevation myocardial infarction; TNI, Troponin I; CK-MB, Creatine Kinase-MB

**Table 2:** Lesion characteristics

		pre propensity-matched analysis			post propensity-matched analysis		
		M-BSKT	PS	*P* Value	M-BSKT	PS	*P* Value
		n = 40	n = 80		n = 34	n = 34	
Location	LAD-D1	27 (67.5%)	56 (70.0%)	0.481	26 (76.5%)	24 (70.6%)	0.741
	LAD-LCX	7 (17.5%)	13 (16.3%)		6 (17.6%)	8 (23.5%)	
	LCX-OM	2 (5.0%)	7 (8.8%)		2 (5.9%)	1 (2.9%)	
	LAD-RI	3 (7.5%)	1 (1.3%)		0	1 (2.9%)	
	RCA-PD	1 (2.5%)	3 (3.8%)		0	0	
Medina	1.1.1	31 (77.5%)	63 (78.8%)	1	26 (76,5%)	25 (73.5%)	0.846
	1.0.1	5 (12.5%)	9 (11.25%)		4 (11.8%)	6 (17.6%)	
	0.1.1	4 (10.0%)	8 (10.0%)		4 (11.8%)	3 (8.8%)	

Values are n(%). LAD, left anterior descending; D, diagonal artery; LCX, left circumflex; OM,obtuse marginal branch; RCA,right coronary artery; RI, ramus intermedius artery; PD, posterior descending artery

### Procedural outcomes

**Fig. 3:**
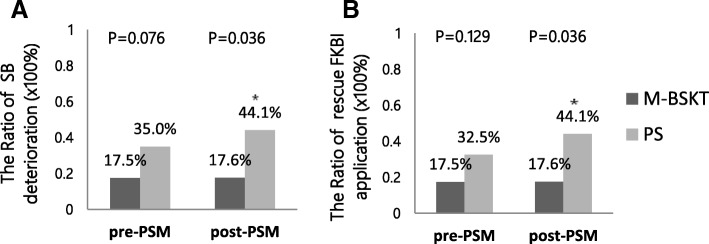
**a** The Ratio of SB deterioration. The *P*-values of the incidence of immediate SB deterioration between the 2 groups (pre-PSM and post-PSM). **b** The Ratio of rescue FKBI application. The *P*-values of the rescue FKBI application between the 2 groups (pre-PSM and post-PSM). PSM: propensity-matched analysis. FKBI: final kissing balloon inflation. * means *P* < 0.05

The immediate procedural and clinical outcomes are displayed in Table 3. The operation successful rate was 100% in each group. The study showed no statistically significant clinical impact of SB deterioration [M-BSKT *n* = 7(17.5%); PS *n* = 28(35.0%); *P* = 0.076] in 2 groups (Fig. 3a). The rates of SB TIMI flow < 3 were 7.5% and 15%[*P* = 0.38], severe SB ostial pinching (≥90%) were 10% and 17.5% [*P* = 0.412], in the M-BSKT versus the PS groups, respectively. Besides, 2 patients’ SB occluded immediately in PS, and the other patients whose SB damaged underwent FKBI [M-BSKT *n* = 7(17.5%); PS *n* = 26(32.5%); *P* = 0.129] (Fig. 3b). SB flow was restored spontaneously in 2 groups [M-BSKT n = 7(17.5%); PS *n* = 23(28.8%); *P* = 0.263]. The use of bailout SB stenting was 2.5% of the PS group [n = 2; *P* = 0.552]. Compared with M-BSKT, 5 patients’SB in PS were inevitable occluded despite all SB had jailed wire, even though 3 of them underwent FKBI [M-BSKT *n* = 0; PS *n* = 5(6.3%)); *P* = 0.168]. All the wires and balloons were not entrapped during the operations.

**Table 3:** Outcomes

	pre propensity-matched analysis			post propensity-matched analysis		
	M-BSKT	PS	*P* Value	M-BSKT	PS	*P* Value
	*n* = 40	*n* = 80		*n* = 34	*n* = 34	
immediate procedural and clinical outcomes						
Device and procedural success	40 (100.0%)	80 (100.0%)	1	34 (100.0%)	34 (100.0%)	1
Main-vessel TIMI flow 3 after procedure	40 (100.0%)	80 (100.0%)	1	34 (100.0%)	34 (100.0%)	1
Side-branch deterioration	7 (17.5%)	28 (35.0%)	0.076	6 (17.6%)	15 (44.1%)	0.036
SB TIMI flow <3	3 (7.5%)	12 (15%)	0.38	3 (8.8%)	5 (14.7%)	0.709
SB ostial pinching≥90%	4 (10%)	14 (17.5%)	0.412	3 (8.8%)	10 (29.4%)	0.062
SB occlusion immediately	0	2 (2.5%)	0.552	0	0	NS
rescue FKBI	7 (17.5%)	26 (32.5%)	0.129	6 (17.6%)	15 (44.1%)	0.036
Bailout stenting	0	2 (2.5%)	0.552	0	0	NS
Revascularization	7 (17.5%)	23 (28.8%)	0.263	6 (17.6%)	13 (38.2%)	0.104
SB loss	0	5 (6.3%)	0.168	0	2 (5.9%)	0.492
Wire or balloon damaged	0	0	NS	0	0	NS
The prognosis after 12 months						
Stable condition	37 (92.5)	73 (91.3%)	0.558	31 (91.2%)	32 (94.1%)	0.613
Rehospitalization for unstable angina	3 (7.5%)	4 (5.0%)		3 (8.8%)	1 (2.9%)	
MACEs	0	3 (3.8%)		0	1 (2.9%)	

Values are n(%).TIMI, thrombolysis in myocardial infarction; FKBI, final kissing-balloon inflation; MACEs, major adverse cardiac events

### Clinical outcomes at 12 months

**Fig. 4:**
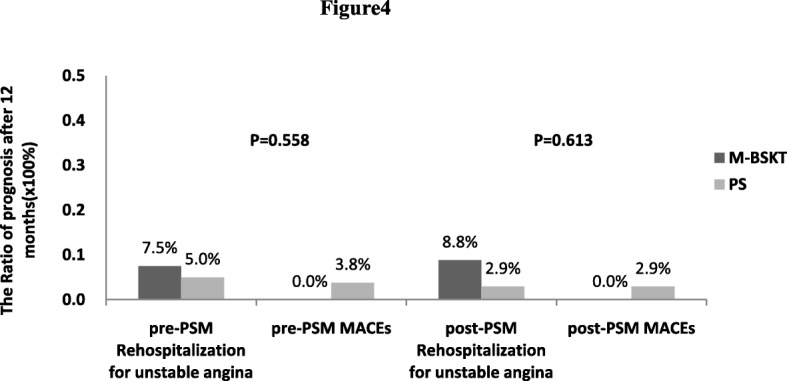
Clinical outcomes at 12 months. PSM = propensity-matched analysis. Before PSM, 12 months clinical outcomes about rehospitalization for unstable angina and MACEs did not differ between patients treated with M-BSKT and PS. The prognosis at 12 months were similar between 2 groups after PSM

Long-term clinical outcomes did not differ between patients treated with M-BSKT and PS at 12 months [*P* = 0.558]. The cumulative rehospitalization for unstable angina was similar between M-BSKT [3(7.5%)] and PS [n=4(5.0%)]. The cumulative 1-year MACEs was only occurred in PS group [n=3(3.8%)] (Table 3 and Fig. 4).

### Propensity score matching

We conducted propensity score matching by using R package matching and matched patients within a ± 0.2 caliper range. The following 15 preoperative variables were considered in the stepwise logistic regression multivariable analysis: male, age, hypertension, diabetes mellitus, hyperlipidemia, smoke, prior CABG, prior PCI, prior MI, chronic kidney disease, diagnosis at PCI, Troponin I (TNI), Creatine Kinase-MB (CK-MB), the location of bifurcation lesions and Medina classification.

A matched pair of 34 M-BSKT and 34 PS patients was identified. Patient characteristics were well matched between the 2 groups in Tables 1 and 2.After propensity score matching, the incidence of immediate SB deterioration was significant lower in M-BSKT than PS [M-BSKT *n* = 6(17.6%); PS *n* = 15(44.1%); *P* = 0.036] (Table 3; Fig. 3a). Among them, there was no distinction about TIMI flow deterioration [M-BSKT *n* = 3(8.8%); PS *n* = 5(14.7%);*P* = 0.709] and ostial pinching [M-BSKT n = 3(8.8%);PS *n* = 10(29.4%);*P* = 0.062] between two groups. The application of rescue FKBI in M-BSKT was significant lower than PS [M-BSKT n = 6(17.6%); PS *n* = 15(44.1%); *P* = 0.036](Fig. 3b). In spite of all the patients who defined as SB deterioration underwent FKBI, 2 patients’ SB were lost in PS group [*n* = 2(5.9%), *P* = 0.492]. The prognosis at 12 months such as rehospitalization for unstable angina [M-BSKT n = 3(8.8%); PS n = 1(2.9%)] and MACEs [M-BSKT *n* = 0; PS n = 1(2.9%)] were similar between the two groups [*P* = 0.613] showed in Table 3 and Fig. 4.

### Subgroup analysis of acute coronary syndrome (ACS)

**Fig. 5:**
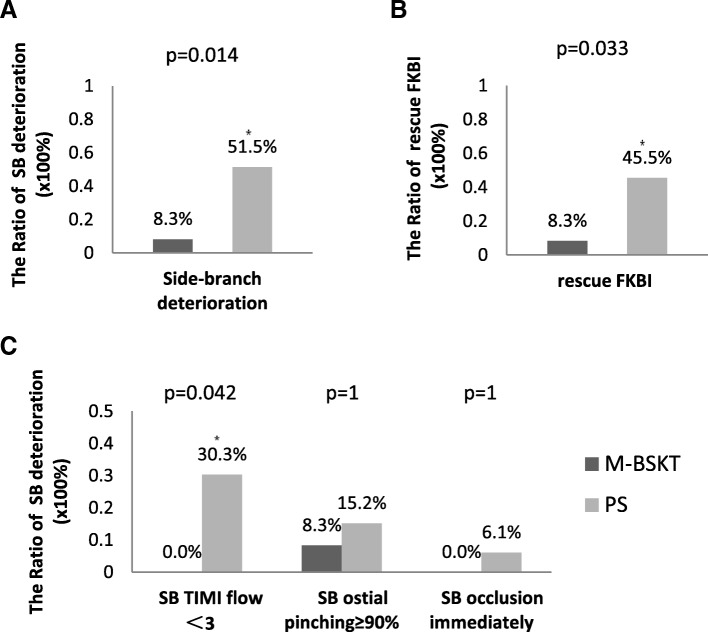
immediate procedural and clinical outcomes of ACS patients. Subgroup analysis of ACS patients: (**a**) The ratio of immediate SB deterioration (**b**) The ratio of rescue FKBI application (**c**) The ratio of the each scenes of SB deterioration between the 2 groups . * means *P* < 0.05

Baseline Characteristics and procedural outcomess of patients with acute coronary syndrome in the two groups were reanalyzed. The ACS patients were all recruited in two subgroups; 1) M-BSKT group (*n* = 12) and 2) PS group (*n* = 33). There were no significant differences in baseline clinical characteristics between the two groups. Angiographic and procedural characteristics of the two groups are shown in Table 4. The immediate SB deterioration in the PS group [*n* = 17(51.5%)] was significant higher compared to M-BSKT group [n = 1(8.3%);*P* = 0.014] (Fig. 5a). All situations of SB deterioration were not identical between two groups (Fig. 5c). The PS group displayed a higher (*P* = 0.042) rate [n = 10(30.3%)] of SB TIMI flow< 3 than M-BSKT. FKBI was more frequently performed in PS group [n = 15(45.5%)] than M-BSKT group [*n* = 1(8.3%);*P* = 0.033] (Fig. 5b). Despite the SB loss in two groups showed no difference, there were 4 (12.1%) patients in ACS patients, which made a big proportion of all patients.

**Table 4:** immediate procedural and clinical outcomes of ACS patients

patients with acute coronary syndrome			
	M-BSKT	PS	*P* Value
	*n* = 12	*n* = 33	
immediate procedural and clinical outcomes			
Device and procedural success	12 (100.0%)	33 (100.0%)	1
Main-vessel TIMI flow3 after procedure	12 (100.0%)	33 (100.0%)	1
Side-branch deterioration	1 (8.3%)	17 (51.5%)	0.014
SB TIMI flow <3	0	10 (30.3%)	0.042
SB ostial pinching≥90%	1 (8.3%)	5 (15.2%)	1
SB occlusion immediately	0	2 (6.1%)	1
rescue FKBI	1 (8.3%)	15 (45.5%)	0.033
Bailout stenting	0	1 (3.0%)	1
Revascularization	1 (8.3%)	13 (39.4%)	0.07
SB loss	0	4 (12.1%)	0.561
Wire or balloon damaged	0	0	NS

Values are n(%).TIMI, thrombolysis in myocardial infarction; FKBI, final kissing-balloon inflation

## Discussion

The present study focus on clinical outcomes between M-BSKT and PS techniques for simple true bifurcation lesions stratified the criteria. The principal findings in this study are as follows: 1) In general, M-BSKT was similar to PS in every aspects except the damage of SB and the rate of FKBI application. 2) In ACS patients, performing M-BSKT was a significant factor to protect SB, especially in preventing the deterioration of TIMI flow and reducing the application of FKBI.

Although the ESC guidelines strongly recommends provisional SB stenting taking into consideration not only immediate but also long-term results of this strategy [22], abrupt closure of the SB may occur after MV stent implantation [12–14]. Meanwhile, SB occlusion occurred more frequently in patients with true bifurcation lesions than in those with non-true bifurcation lesions [23]. In the present clinical trial, the final outcomes of provisional stenting of bifurcation lesions were not associated with significant improvement [15, 24]. In our study, we compared the safety and efficacy of PS and M-BSKT during the treatment of some especial bifurcation lesions. It adopted the criteria of true bifurcation lesions and eliminated the complex bifurcation lesions established by Chen [20]. Based on previous researches, we eliminated complex bifurcation lesions that may get the maximum benefit from 2-stent techniques. Since a proportion of patients with a severe big SB lesions (simple true bifurcation lesions) would remain ischemic after MV stenting, the jailed SB wire or balloon facilitated rewiring of the SB by widening the angle between the MV and SB and prevented SB occlusion. This experiment using SB ostial pinching≥90% by angiography as an indication for SB intervention reduced the unnecessary intervention of SB in the maximum limit. Meanwhile, a bailout stent in side branch was implanted only in case of dissection or significant flow impairment after FKBI.

The construction of the classical stent did not take into consideration vessel tapering in bifurcation lesions and resulted in carina and plaque shift—the main mechanisms of SB compromise [15]. However, the M-BSKT consists of leaving a dilated balloon in SB while implanting a stent in the MV. It impacts less on the bifurcation segment by means of limiting carina and plaque shift towards to SB, which are two major factors responsible for SB compromise [18]. Yohei Numasawa [17] evaluated the configuration of true bifurcation lesion after stent implantation using the jailed balloon technique by three-dimensional optical frequency domain imaging (OFDI) which provided clearer and higher resolution images. It was already indicated clearly that there were no signs of plaque or carina shift into the SB. However, there was little data to support the advantage of this technique. In this case, we clustered data from 2 groups about the SB ostial deteriorations and found out all deteriorations were slightly less when applying M-BSKT than PS. The advantage of M-BSKT that balloon inflated between the time from the stent located to inflated could protect SB more effective in theory.

Based on the date and trends we observed, the implementation of M-BSKT had no significant differences regarding to SB protection in the 2 study groups, but a trend towards superior clinical results in patients treated with M-BSKT. So the propensity score matching was used to identify the diversity between the 2 techniques. Baseline features were well balanced among M-BSKT patients versus PS patients. We noted the difference in immediate procedural and clinical outcomes between the 2 groups in the matched cohort. When propensity score matching was adopted, the effect of SB protection revealed the superior technical advantages of M-BSKT comparing with PS. As a result, the rate of SB flow deterioration and dissection after MV stenting at once was higher in PS. Because of cardiac death or myocardial infarction occurred more frequently in patients with SB occlusion [14], it is conceivable to infer that the inflated balloon of the SB provides a high degree of SB protection in immediate procedural.

According to research results available, jailed wire in SB was associated with the recovery of the occluded SB [14]. What’s more, it indicated that M-BSKT used as a rescue procedure to keep the SB open was superior to PS on the basis of technological advantages, once the branch was occluded. This led to significantly reduced proximal deformation with related malapposed struts and reduced rate of SB ostial stenosis. Jailed balloon in SB could be inflated and FKBI could be applied, as long as the damage of SB flow happened accidentally. In some respect, MB restenosis was higher in the routine FKBI group due to the potential distortion of the MB stent strut [25]. On this premise, it is recommended to avoid deploying FKBI technique. The hypothesis that M-BSKT could decrease the proportion of FKBI implement during operation was justified in this study.

Interestingly, when we conducted subgroup analysis of patients with acute coronary syndrome, the advantages of M-BSKT were showed prominently. It just made a clarity conclusion that M-BSKT may provide more protection of SB. Patients with ACS are likely to have multiple vulnerable plaques that are liable to rupture or shift [26, 27]. According to ACS patients with special bifurcation lesions, M-BSKT may limit thrombosis and plaque shift towards to SB more effectively, which avoid the impact on TIMI flow and reduce the application of FKBI.

As we all know, POT consists of an inflating balloon to the MV reference diameter which makes the malapposition in the stented MV segment completely corrected while maintaining perfect arterial circularity [19]. Though high rates of restenosis and stent thrombosis were still often observed after stenting, POT would provide potential benefits such as partially reducing malapposition and achieving effective modification of physiological anatomy [19, 28]. In this study, POT was used as a favourable step to modify BSKT in order to reduce severe coronary flow problems.

Irrespective of propensity score matching, the findings of 1 year clinical follow-up were similar between the 2 techniques. However, wether M-BSKT will play a safe and effective role for a longer period of time is still unknown. Evaluation of long clinical outcomes of two stent percutaneous intervention strategy for treatment of coronary bifurcation lesions offered advantage over PS, simultaneously [11]. Therefore, with our present data, M-BSKT elicited more advantages than PS applied to simple true bifurcation lesions in the operation procedure.

## Limitations

The first and foremost limitation of this study was that it did not involve a significant population. Also, visual estimation of the diameters was used in this study to dedicate bifurcations, rather than quantitative coronary measurement. It may be not precise for the selection of clinically relevant SB. Previous studies suggested that angiographic visual assessment of jailed SB lesions tended to overestimate the severity of jailed SB lesions compared to functional assessment by FFR. Regrettably, there was no application of FFR in this study. Differences in the angiographic assessment of SB lesion severity can affect the treatment strategy for bifurcation lesions. This manuscript used SB ostial pinching≥90% by angiography as indication for SB intervention that was reduced the unnecessary intervention of SB in the maximum limit. Of note, there was a discrepancy between the assessment of the ischemia-inducibility and plan-to-treat. These variable and discrepant values might have influenced the differences of SB intervention between two groups. Although bifurcation angle was regarded as one of the important factors for bifurcation PCI, we excluded it from the scope of criteria, which may influence the SB-occlusion rate. In addition, fractional flow reserve for evaluating the significance of SB ostial lesions wasn't used before and after the procedure. Because randomization may interfere with the chosen of different bifurcations, the simple true bifurcations of the right coronary artery were rarely contained in our study.

## Conclusions

We demonstrated that, for patients with coronary simple true bifurcation lesions, M-BSKT provides preferable outcomes comparing with the PS after propensity score matching. Because of the existence of vulnerable plaques in ACS patients exist, the advantages of M-BSKT were showed prominently. Our case reported that it appeared to be a reasonable and suitable strategy to reduce the deterioration of SB and the application of FKBI when MV stent was inflated during the operation. Although M-BSKT has been performed, its long-term impact on patients' outcomes cannot be ascertained based on current studies and requires further investigation on the correlation between BSKT and clinical outcomes.

## Abbreviations

ACS: Acute coronary syndrome
CABG: Coronary artery bypass graft
CK-MB: Creatine kinase-MB
FFR: Fractional flow reserve
FKBI: Final kissing balloon inflation
MADEs: Major adverse cardiac events
M-BSKT: Modified balloon-stent kissing technique
MI: Myocardial infarction
MV: Main vessel
NSTEMI: Non-ST-segment elevation myocardial infarction
OFDI: Optical frequency domain imaging
PCI: Percutaneous coronary intervention
POT: Proximal optimization technique
PS: Provisional stenting
SB: Side branch
STEMI: ST-segment elevation myocardial infarction
TLR: Target lesion revascularization
TNI: Troponin I
TVR: Target vessel revascularization
